# Anxiety levels among medical students and healthcare workers during Covid-19 pandemic: Bulgaria sample

**DOI:** 10.1192/j.eurpsy.2025.1030

**Published:** 2025-08-26

**Authors:** E. Papazov, P. Marinov, A. Chankov

**Affiliations:** 1 Mental Health Center Prof. N. Shipkovenski; 2 SOFIA UNIVERSITY ST. KLIMENT OHRIDSKI, Sofia; 3 Trakia University, Stara Zagora, Bulgaria

## Abstract

**Introduction:**

The Covid-19 pandemic has been an integral part of our lives since late 2019, bringing significant changes to the way we live. These changes have brought with them a wave of pandemic-related psychological distress including fear, anxiety, feelings of threat. The Covid-19 pandemic presented several global challenges to the functioning of the health system in general and to medical personnel in particular. Unfortunately, the discussion on how staff in health facilities deal with pandemic-associated distress has remained in the background.

**Objectives:**

The main aim of our study was to investigate the impact of the COVID-19 pandemic on the mental health of healthcare workers and trainees and specifically on the development of anxiety symptoms. We aimed to track levels of anxiety among physicians, health care professionals, and medical students, and their relationship to various sociodemographic indicators-gender, age, marital status, education and occupation, familial predisposition to anxiety disorders, and previous consultation with a psychologist/psychiatrist.

**Methods:**

The study was led among fifth and sixth year medical students in Sofia university and Trakia university and also doctors and nurses working in hospitals in Sofia and Stara Zagora - MHC “Prof. Н. Shipkovensky”, University Hospital “N.I Pirogov”, University Hospital “Prof. Dr. Stoyan Kirkovich”. The study used a questionnaire created by the researchers and the Coronavirus Anxiety Scale by Lee and the Covid-19 anxiety syndrome scale by Nikcevic and Spada.

**Results:**

The mean age of the study participants was 28.21±10.11 years ranging from 22 to 68. Majority of the study participants (72.3%) were students followed by doctors with 19.8% and nurses with 5.6%. After statistical analysis of the data, there was a linear relationship between having a close friend or relative who had died or had a serious illness due to Covid-19 infection and having marked anxiety and fear for the health of loved ones. In this group frequent checking for coronavirus symptoms, both in the respondents themselves and by them to their friends and relatives was observed more often. Pre-pandemic visits to a psychologist/psychiatrist for anxiety symptoms appeared to be a predictor of avoidance behaviour in respondents e.g.not using public transport to reduce the risk of infection.

**Conclusions:**

Having a close friend or relative who had a severe infection with Covid-19 and pre-pandemic visits to a mental health professional were predictors of increased pandemic-related anxiety and avoidance behavior among health care professionals and medical students. Due to the high prevalence of this mental health problem among medical students and frontline health workers, it is suggested that healthcare institutions provide mental health services for these working groups in order to appropriately manage anxiety.

**Disclosure of Interest:**

None Declared

